# Analyzing secretory proteins in human dermal fibroblast‐conditioned medium for angiogenesis: A bioinformatic approach

**DOI:** 10.1111/srt.13568

**Published:** 2024-01-10

**Authors:** Sang Bum Suh, Ji Youn Suh, Sung Bin Cho

**Affiliations:** ^1^ BNV Biolab Seoul South Korea; ^2^ Yonsei Seran Dermatology and Laser Clinic Seoul South Korea

**Keywords:** angiogenesis, bioinformatics, dermal fibroblast‐conditioned media, human dermal fibroblast, proteomic analysis, secretory protein

## Abstract

**Background:**

The conditioned medium from human dermal fibroblasts (dermal fibroblast‐conditioned medium; DFCM) contains a diverse array of secretory proteins, including growth factors and wound repair‐promoting proteins. Angiogenesis, a crucial process that facilitates the infiltration of inflammatory cells during wound repair, is induced by a hypoxic environment and inflammatory cytokines.

**Methods:**

In this study, we conducted a comprehensive bioinformatic analysis of 337 proteins identified through proteomics analysis of DFCM. We specifically focused on 64 DFCM proteins with potential involvement in angiogenesis. These proteins were further classified based on their characteristics, and we conducted a detailed analysis of their protein–protein interactions.

**Results:**

Gene Ontology protein classification categorized these 64 DFCM proteins into various classes, including metabolite interconversion enzymes (*N* = 11), protein modifying enzymes (*N* = 10), protein‐binding activity modulators (*N* = 9), cell adhesion molecules (*N* = 6), extracellular matrix proteins (*N* = 6), transfer/carrier proteins (*N* = 3), calcium‐binding proteins (*N* = 2), chaperones (*N* = 2), cytoskeletal proteins (*N* = 2), RNA metabolism proteins (*N* = 1), intercellular signal molecules (*N* = 1), transporters (*N* = 1), scaffold/adaptor proteins (*N* = 1), and unclassified proteins (*N* = 9). Furthermore, our protein–protein interaction network analysis of DFCM proteins revealed two distinct networks: one with medium confidence level interaction scores, consisting of 60 proteins with significant connections, and another at a high confidence level, comprising 52 proteins with significant interactions.

**Conclusions:**

Our bioinformatic analysis highlights the presence of a multitude of secretory proteins in DFCM that form significant protein–protein interaction networks crucial for regulating angiogenesis. These findings underscore the critical roles played by DFCM proteins in various stages of angiogenesis during the wound repair process.

## 1 INTRODUCTION

The significance of human dermal fibroblast (HDF)‐derived secretomes in promoting wound repair has been extensively explored, revealing their pivotal roles in this process.^1^, ^2^, ^3^, ^4^, ^5^ These secretomes encompass a spectrum of growth factors, including epidermal growth factor (EGF), basic fibroblast growth factor (FGF), FGF‐7, vascular endothelial growth factor (VEGF), platelet‐derived growth factor (PDGF), insulin‐like growth factor binding proteins (IGFBP)‐4 and IGFBP‐6, transforming growth factor (TGF)‐β1, hepatocyte growth factor (HGF), and keratinocyte growth factor.^3^, ^6^, ^7^ Remarkably, studies using growth factor G1 arrays have indicated that HDF‐derived secretomes induce other cell types to secrete markedly higher amounts of growth factors compared to control cells.^7^

To delve into HDF‐derived secretomes, researchers have employed proteomic approaches to analyze dermal fibroblast‐conditioned medium (DFCM).^3^, ^5^ In our previous work, our study group identified 337 secretory proteins from DFCM preparations.^5^ Bioinformatic analyses further unveiled intricate protein–protein interaction networks within these secretomes, closely associated with wound repair and hair regeneration.^5^ DFCM proteins play a pivotal role in activating signal transduction mediators, enhancing wound repair via various signaling pathways, including those involving EGF receptor, FGF, integrin, Wnt, and TGF‐β.^5^, ^8^, ^9^ Moreover, they orchestrate actin and myosin cytoskeletal dynamics through Rho guanosine triphosphatases (GTPases) during the wound healing process.^5^, ^10^, ^11^ Interestingly, DFCM preparations have been found to contain several peptide factors and signaling pathways associated with hair regeneration.^5^ Additionally, DFCM proteins intended for wound repair can induce hair regeneration by expediting mesenchymal cell proliferation and neovascularization.^12^, ^13^, ^14^

In the context of wound repair, the formation of new blood vessels, driven by a hypoxic environment and inflammatory cytokines, is a crucial process that facilitates the infiltration of inflammatory cells, particularly neutrophils and macrophages.^15^ Macrophages themselves produce an array of secretory factors that coordinate angiogenic responses and modulate the formation of new blood vessels.^15^, ^16^, ^17^ In the loose extracellular matrix, angiogenesis is regulated by key factors including hypoxia‐inducible factor (HIF)‐1α, angiopoietin‐2, stromal cell‐derived factor‐1, VEGF, FGF, and heparan sulfate proteoglycans.^15^, ^18^, ^19^, ^20^

In this study, our objective was to perform a comprehensive bioinformatic analysis of secretory proteins in HDF‐derived DFCM to elucidate their potential role in angiogenesis. We conducted bioinformatic analysis on the 337 proteins identified in DFCM through previous proteomic analysis.^5^ Subsequently, we selected DFCM proteins with potential involvement in angiogenesis and classified them based on their characteristics, followed by an in‐depth analysis of their protein–protein interactions. This investigation aims to shed light on the contributions of DFCM proteins to the critical process of angiogenesis.

## 2 MATERIALS AND METHODS

### 2.1 Preparation of DFCM for proteomic analysis

In this study, a total of 337 secretory proteins were subjected to bioinformatic analysis to explore their relevance to angiogenesis.^5^ These 337 proteins had been previously identified through quantitative liquid chromatography tandem mass spectrometry of DFCM preparations, employing matrix‐assisted laser desorption/ionization tandem time‐of‐flight.^5^ The DFCM preparation was obtained from neonatal HDFs (NHDF‐Neo; Lonza, Walkersville, MD, USA).^5^ Specifically, HDFs at passage 5 were cultivated in 175‐cm2‐T flasks, using Opti‐modified Eagle's medium (Gibco BRL, Rockville, MD, USA) supplemented with 2‐mM GlutaMAX, 2‐ng/mL recombinant human EGF, and 2‐ng/mL recombinant human FGF (all purchased from Lonza), without the addition of fetal bovine serum to create low‐serum culture conditions.^5^ The cells were incubated at 37°C in a 5% CO_2_ incubator for 24 h, and the collected medium constituted the DFCM.^5^

### 2.2 Bioinformatic analysis of DFCM proteins

**FIGURE 1:**
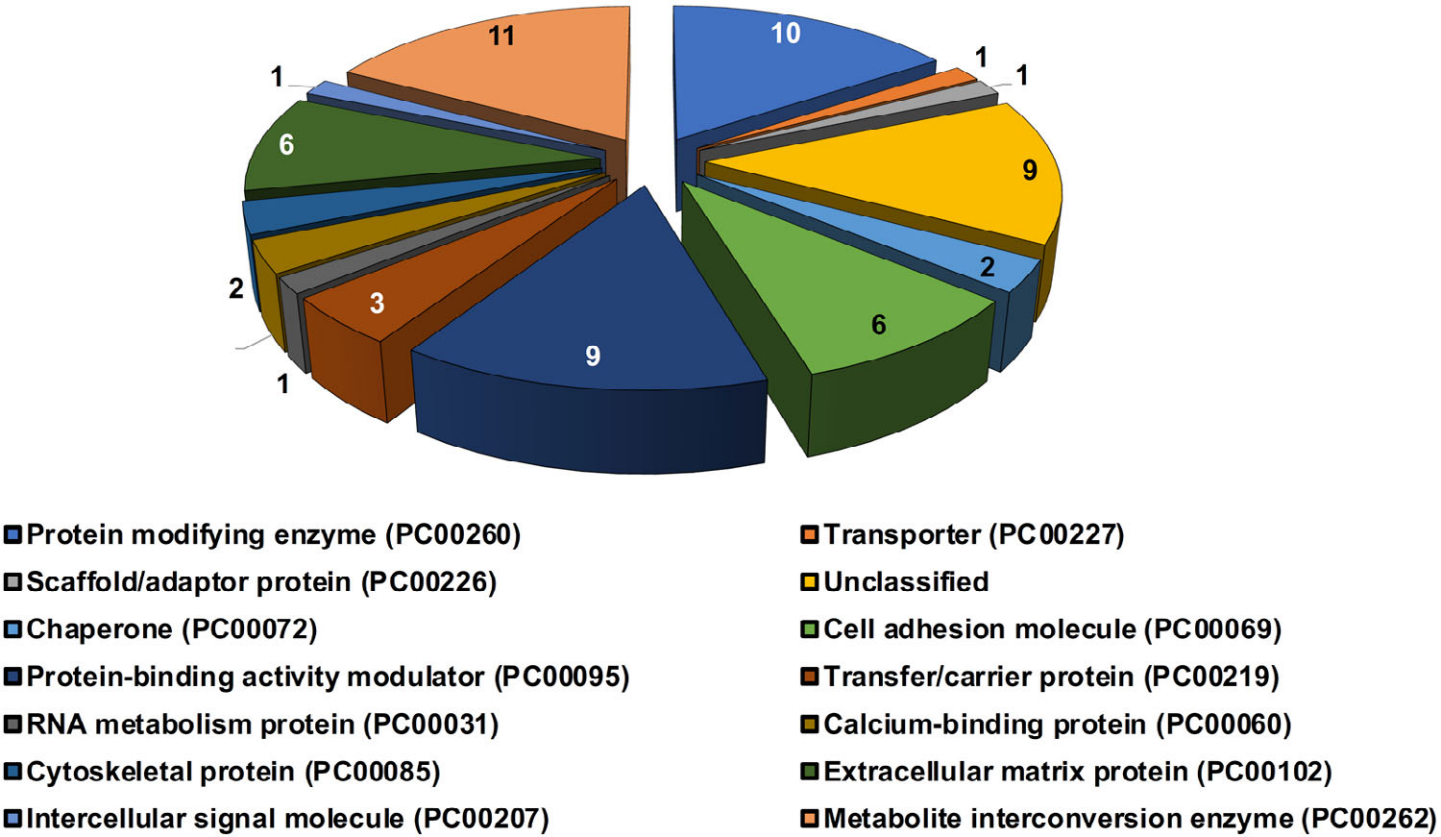
Gene Ontology (GO) protein classification of DFCM proteins involved in angiogenesis regulation. GO categorized 64 DFCM proteins, which were bioinformatically linked to angiogenesis regulation, based on protein classes.

To delineate the functions of the 337 proteins, their known roles were examined through the UniProt Database. From this pool, 64 proteins, showing potential involvement in angiogenesis, were singled out for further scrutiny.^5^ These initially selected proteins were subsequently categorized based on their protein classes using the Protein Analysis Through Evolutionary Relationships (PANTHER™) Classification System v.17.0 (http://pantherdb.org/).^21^ The protein classes for the 64 proteins encompassed a wide spectrum, including RNA metabolism protein (PC00031), calcium‐binding protein (PC00060), cell adhesion molecule (PC00069), chaperone (PC00072), cytoskeletal protein (PC00085), protein‐binding activity modulator (PC00095), extracellular matrix protein (PC00102), intercellular signal molecule (PC00207), transfer/carrier protein (PC00219), transporter (PC00227), scaffold/adaptor protein (PC00226), protein modifying enzyme (PC00260), metabolite interconversion enzyme (PC00262), and unclassified (Figure 1). In addition to this classification, the 64 proteins were further subjected to an assessment of their protein–protein interaction characteristics. This analysis was conducted utilizing the Search Tool for Retrieval of Interacting Genes/Proteins (STRING®) v.11.5 protein interaction network (http://www.string‐db.org/) at four different confidence levels: low, medium, high, and highest.^22^

## 3 RESULTS

### 3.1 Gene Ontology protein classification of DFCM proteins for angiogenesis

***RNA metabolism protein***. Within the category of RNA metabolism protein, nucleolin (accession number, P19338; score, 2.25; coverage, 6%) emerged as a notable constituent of DFCM preparation.

***Calcium‐binding protein***. In the calcium‐binding protein category of DFCM, annexin A2 (accession number, P07355; score, 39.81; coverage, 45%) and annexin A1 (accession number, P04083; score, 14.03; coverage, 25%) were identified.

***Cell adhesion molecule***. The category of cell adhesion molecules comprised six proteins in DFCM, including thrombospondin‐1 (accession number, P07996; score, 56.07; coverage, 12%), laminin subunit α4 (accession number, Q16363; score, 37.29; coverage, 18%), perlecan (accession number, P98160; score, 30.81; coverage, 3%), Thy‐1 membrane glycoprotein (accession number, P04216; score, 9.45; coverage, 15%), TGF‐β‐induced protein ig‐h3 (accession number, Q15582; score, 8.86; coverage, 6%), and periostin (accession number, Q15063; score, 4.23; coverage, 6%).

***Chaperone***. The chaperone category includes heat shock protein (HSP) β1 (accession number, P04792; score, 22.35; coverage, 28%) and nucleophosmin (accession number, P06748; score, 10.87; coverage, 14%) were categorized into chaperone in DFCM.

***Cytoskeletal protein***. The cytoskeletal protein category encompasses plectin (accession number, Q15149; score, 6.84; coverage, 1%) and myosin‐9 (accession number, P35579; score, 5.54; coverage, 4%) were categorized into cytoskeletal protein in DFCM.

***Protein‐binding activity modulator***. Nine proteins were classified as protein‐binding activity modulators, including plasminogen activator inhibitor (accession number, P05121; score, 313.51; coverage, 67%), complement C3 (accession number, P01024; score, 64.11; coverage, 9%), pigment epithelium‐derived factor (accession number, P36955; score, 29.39; coverage, 28%), α2‐macroglobulin (accession number, P01023; score, 29.08; coverage, 6%), plasma protease C1 inhibitor (accession number, P05155; score, 23.19; coverage, 12%), complement C4‐A (accession number, P0C0L4; score, 8.51; coverage, 4%), ribonuclease inhibitor (accession number, P13489; score, 7.16; coverage, 6%), thyroxine‐binding globulin (accession number, P05543; score, 6.21; coverage, 7%), and Ras GTPase‐activating‐like protein (IQGAP1; accession number, P46940; score, 4.51; coverage, 2%).

***Extracellular matrix protein***. Six DFCM proteins were categorized as extracellular matrix proteins, including collagen α‐2 (I) chain (accession number, P08123; score, 236.23; coverage, 50%), fibronectin (accession number, P02751; score, 203.32; coverage, 29%), collagen α‐1 (I) chain (accession number, P02452; score, 177.84; coverage, 42%), laminin subunit β‐1 (accession number, P07942; score, 63.77; coverage, 18%), collagen α‐1 (V) chain (accession number, P20908; score, 26.15; coverage, 6%), and collagen α‐2 (IV) chain (accession number, P08572; score, 2.35; coverage, 2%).

***Intercellular signal molecule***. Semaphorin‐5A (accession number, Q13591; score, 3.51; coverage, 2%) was found in DFCM as an intercellular signal molecule.

***Transfer/carrier protein***. Hemoglobin subunit α (accession number, P05090; score, 25.79; coverage, 33%), apolipoprotein A1 (accession number, P02647; score, 22.41; coverage, 10%), and apolipoprotein D (accession number, P05090; score, 4.29; coverage, 22%) were categorized as transfer/carrier proteins in DFCM.

***Transporter***. Chloride intracellular channel protein 4 (accession number, Q9Y696; score, 5.04; coverage, 36%), found among DFCM secretory proteins, was categorized as a transporter protein.

***Scaffold/adaptor protein***. EMILIN1 (accession number, Q9Y6C2; score, 5.2; coverage, 4%), an elastic microfibril‐associated protein identified in DFCM, was classified as a scaffold/adaptor protein.

***Protein modifying enzyme***. Ten DFCM proteins, including hemopexin (accession number, P00734; score, 257.66; coverage, 45%), 72‐kDa type IV collagenase (accession number, P08253; score, 53.62; coverage, 31%), interstitial collagenase (accession number, P03956; score, 35.95; coverage, 31%), prothrombin (accession number, P00734; score, 14.75; coverage, 5%), aminopeptidase N (accession number, P15144; score, 12.27; coverage, 12%), haptoglobin (accession number, P00738; score, 6.33; coverage, 10%), coagulation factor X (accession number, P00742; score, 6.22; coverage, 4%), adipocyte enhancer‐binding protein 1 (accession number, Q8IUX7; score, 5.24; coverage, 4%), complement C1r subcomponent (accession number, P00736; score, 4.65; coverage, 12%), and bone morphogenetic protein (BMP) 1 (accession number, P13497; score, 2.48; coverage, 3%), were categorized as protein modifying enzymes.

***Metabolite interconversion enzyme***. Eleven DFCM proteins, including lactadherin (accession number, Q08431; score 32.65; coverage 47%), peroxiredoxin‐1 (accession number, Q06830; score 27.63; coverage 37%), glyceraldehyde‐3‐phosphate dehydrogenase (accession number, P04406; score 26.39; coverage 44%), peroxiredoxin‐2 (accession number, P32119; score 18.43; coverage 19%), ectonucleotide pyrophosphatase/phosphodiesterase family member 2 (accession number, Q13822; score 15.18; coverage 12%), macrophage migration inhibitory factor (accession number, P14174; score 10.88; coverage 18%), peroxidasin homolog (accession number, Q92626; score 8.1; coverage 8%), peroxiredoxin‐4 (accession number, Q13162; score 6.68; coverage 12%), peroxiredoxin‐6 (accession number, P30041; score 6.53; coverage 42%), N(G), N(G)‐dimethylarginine dimethylaminohydrolase 1 (DDAH1; accession number, O94760; score 2.53; coverage 13%), and arginase‐1 (accession number, P05089; score 0; coverage 9%), were categorized as metabolite interconversion enzymes.

***Unclassified***. Among the 64 selected DFCM proteins, nine were unclassified. These include keratin, type II cytoskeletal 1 (accession number, P04264; score, 203.46; coverage, 57%), clusterin (accession number, P10909; score, 52.26; coverage, 29%), decorin (accession number, P07585; score, 38.04; coverage, 19%), vinculin (accession number, P18206; score, 36; coverage, 21%), fibulin‐1 (accession number, P23142; score, 31.3; coverage, 14%), junction plakoglobin (accession number, P14923; score, 28.11; coverage, 11%), extracellular matrix protein 1 (accession number, Q16610; score, 28.1; coverage, 20%), receptor of activated protein C kinase 1 (accession number, P63244; score, 3.57; coverage, 9%), and ras‐related protein Rab‐10 (accession number, P61026; score, 1.9; coverage, 11%).

### 3.2 Protein–protein interaction network analysis of DFCM proteins for angiogenesis

**FIGURE 2:**
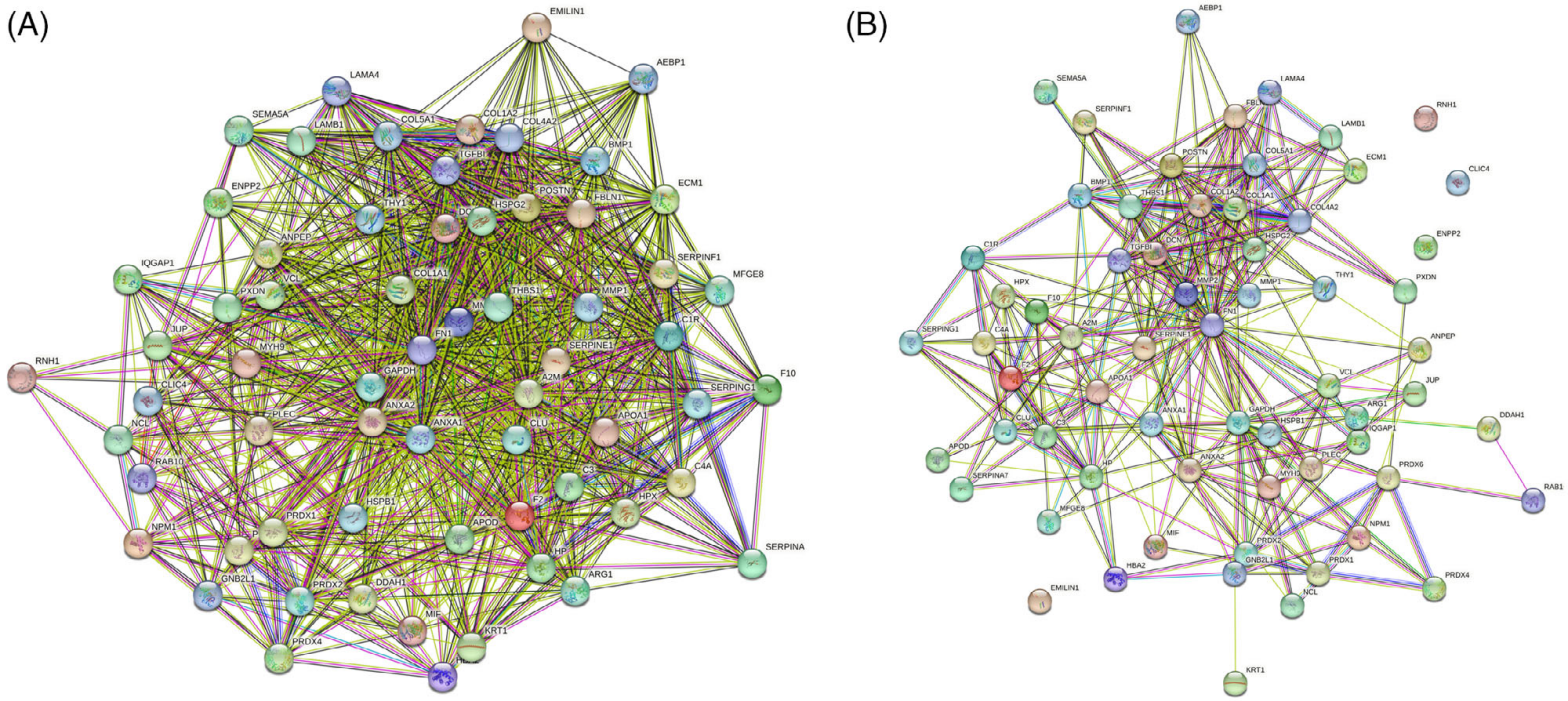
Protein–protein interaction network analysis of 64 DFCM proteins in angiogenesis regulation. (A) At a low confidence level of interaction score (0.15), a single protein–protein interaction network consisting of 64 proteins with significant connections was observed. (B) At a medium confidence level of interaction score (0.4), a single protein–protein interaction network consisting of 60 proteins with significant connections was observed.

At a low confidence level (0.15) of interaction scores, a single protein–protein interaction network encompassed all 64 proteins, revealing notable connections among them (Figure 2A). Meanwhile, when the confidence level was set to medium (0.4) for interaction scores, a single protein–protein interaction network comprised 60 proteins with significant connections (Figure 2B). Notably, four proteins, namely EMILIN1, ribonuclease inhibitor, chloride intracellular channel protein 4, and ectonucleotide pyrophosphatase/phosphodiesterase family member 2, exhibited limited interactions.

**FIGURE 3:**
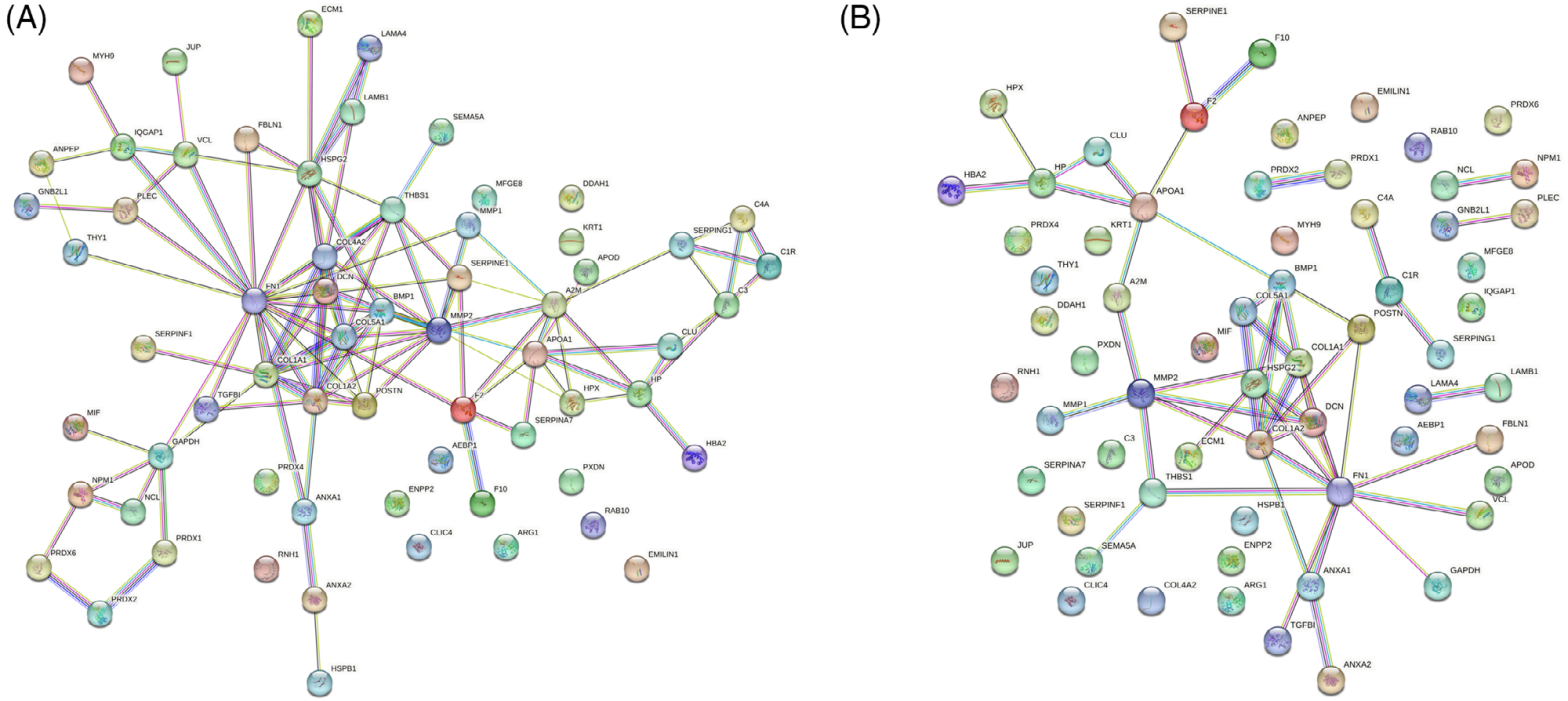
Protein–protein interaction network analysis of 64 DFCM proteins in angiogenesis regulation. (A) At a high confidence level of interaction score (0.7), a single protein–protein interaction network consisting of 52 proteins with significant connections was observed. (B) At the highest confidence level of interaction score (0.9), six protein‐protein interaction networks consisting of 39 proteins with significant connections was observed.

Upon raising the confidence level to a high setting (0.7) for interaction scores, a single protein–protein interaction network involving 52 proteins with significant connections was identified (Figure 3A). In contrast, 12 proteins, including EMILIN1, ribonuclease inhibitor, chloride intracellular channel protein 4, ectonucleotide pyrophosphatase/phosphodiesterase family member 2, peroxiredoxin‐4, adipocyte enhancer‐binding protein 1, arginase‐1, peroxidasin homolog, ras‐related protein Rab‐10, N(G), N(G)‐dimethylarginine dimethylaminohydrolase 1, lactadherin, and keratin, type II cytoskeletal 1, lacked significant interactions with other proteins.

Furthermore, at the highest confidence level (0.9) for interaction scores, six interaction network groups were identified, involving a total of 39 DFCM proteins (summarized in Table 1 and Figure 3B). Notably, 25 proteins within this dataset did not exhibit significant interactions with other proteins.

**TABLE 1:** Six distinctive protein–protein interaction network groups comprising 39 proteins among the 64 DFCM proteins involved in angiogenesis regulation at a confidence score of 0.9.

Group	Accession	Description	Score	Coverage (%)
1	P05121	Plasminogen activator inhibitor 1	313.51	67
	P00734	Hemopexin	257.66	45
	P08123	Collagen α‐2 (I) chain	236.23	50
	P02452	Collagen α‐1 (I) chain	177.84	42
	P02751	Fibronectin	203.32	29
	P07996	Thrombospondin‐1	56.07	12
	P08253	72 kDa type IV collagenase	53.62	31
	P10909	Clusterin	52.26	29
	P07355	Annexin A2	39.81	45
	P07585	Decorin	38.04	19
	P18206	Vinculin	36	21
	P03956	Interstitial collagenase	35.95	31
	P23142	Fibulin‐1	31.3	14
	P98160	Perlecan	30.81	3
	P01023	α‐2‐macroglobulin	29.08	6
	Q16610	Extracellular matrix protein 1	28.1	20
	P04406	Glyceraldehyde‐3‐phosphate dehydrogenase	26.39	44
	P20908	Collagen α‐1(V) chain	26.15	6
	P05090	Hemoglobin subunit α	25.79	33
	P02647	Apolipoprotein A1	22.41	10
	P00734	Prothrombin	14.75	5
	P04083	Annexin A1	14.03	25
	Q15582	TGF‐β‐induced protein ig‐h3	8.86	6
	P00738	Haptoglobin	6.33	10
	P00742	Coagulation factor X	6.22	4
	Q15063	Periostin	4.23	6
	Q13591	Semaphorin‐5A	3.51	2
	P13497	Bone morphogenetic protein 1	2.48	3
2	P05155	Plasma protease C1 inhibitor	23.19	12
	P0C0L4	Complement C4‐A	8.51	4
	P00736	Complement C1r subcomponent	4.65	12
3	Q06830	Peroxiredoxin‐1	27.63	37
	P32119	Peroxiredoxin‐2	18.43	19
4	P06748	Nucleophosmin	10.87	14
	P19338	Nucleolin	2.25	6
5	Q15149	Plectin	6.84	1
	P63244	Receptor of activated protein C kinase	3.57	9
6	P07942	Laminin subunit β‐1	63.77	18
	Q16363	Laminin subunit α‐4	37.29	18

## 4 DISCUSSION

In this study, we selected 64 proteins out of 337 DFCM proteins that were identified through bioinformatics as potentially involved in angiogenesis regulation. Subsequently, utilizing Gene Ontology protein classification, we categorized these 64 DFCM proteins based on their respective protein classes. Additionally, our in‐depth protein–protein interaction network analysis unveiled significant networks among the DFCM proteins. Nucleolin, a major nucleolar protein in proliferating cells, plays a multifaceted role encompassing preribosomal RNA transcription, ribosome assembly, chromatin organization, microRNA processing, RNA and DNA metabolism, ribosome biogenesis, cell division, proliferation, response to stress, regulation of apoptosis, and angiogenesis and lymphangiogenesis.^23^, ^24^ Annexin A2 plays a pivotal role in wound repair by regulating hemostasis, coordinating cytoskeletal organization and membrane repair, and initiating angiogenesis. Additionally, annexin A2's fibrinolytic property promotes the remodeling of the provisional matrix during wound repair.^25^, ^26^ Annexin A1, however, has been shown to enhance reparative angiogenesis and protect against neuronal injury in ischemic tissue.^27^ It is also involved in the apoptosis of inflammatory cells, efferocytosis and phagocytosis of apoptotic cells, pathogens, and debris, as well as the resolution of the inflammatory tissue environment.^28^ Previous research has demonstrated that annexin A1 treatment promotes the angiogenesis process and mitigates exacerbated inflammation, ultimately improving tissue regeneration following skin grafting.^29^

Thrombospondin‐1 is recognized as an endogenous inhibitor of angiogenesis, as it inhibits endothelial cell migration and the angiogenic response to various stimuli while inducing apoptosis in endothelial cells.^30^ Furthermore, thrombospondin‐1 inhibits tumor progression through the suppression of tumor cell proliferation via TGF‐β activation.^31^ Laminin subunit α4 exhibits a high affinity for integrin ligands, mediating endothelial cell‐laminin subunit α4 interaction, which is crucial for angiogenesis.^32^ Perlecan, a basement membrane‐specific heparan sulfate proteoglycan core protein, is incorporated into the basement membrane of vessels during angiogenesis and is involved in maintaining the cutaneous subendothelial basement membrane. Perlecan also promotes angiogenesis in conjunction with FGF‐2.^33^

Thy‐1, also known as CD90, is a glycosyl phosphatidyl inositol‐anchored protein with implications in angiogenesis and vascular perfusion during wound repair.^34^ Furthermore, Thy‐1‐positive fibroblasts within the wound environment have been shown to enhance tissue contraction and fibrotic activity.^35^ TGF‐β‐induced protein ig‐h3, which is a collagen‐binding matrix protein, has been found to be essential in the sprouting and lumen formation of endothelial cells during angiogenesis, in conjunction with other fibroblast‐derived matrix proteins, including collagen α1, procollagen C‐endopeptidase enhancer 1, and IGFBP‐7.^36^ While the precise role of TGF‐β‐induced protein ig‐h3 has not been fully elucidated, previous research suggests that it may play an important role in the matrix interactions of endothelial cells during angiogenesis.^36^, ^37^ Periostin is a multifunctional glycoprotein of the extracellular matrix secreted by mesenchymal cells.^38^ It exhibits high expression under pathological conditions and has been linked to wound repair, inflammation, fibrosis, angiogenesis, tissue remodeling, and regeneration, as well as bone formation and vascular calcification.^38^ During angiogenesis, periostin activates the extracellular signal‐regulated kinases (Erk)/VEGF pathway and interacts with the αvβ3 integrin, enhancing the adhesion and migration of endothelial cells.^39^

HSP‐β1, also known as HSP27, acts as a chaperone for VEGF and regulates its secretion, thus playing a role in angiogenesis.^40^ Nucleophosmin, an endogenous nucleolar protein belonging to the histone chaperone family, has been associated with increased master regulators for angiogenesis when stimulated in endothelial cells, including VEGF, HGF, FGF‐2, PDGF, stromal‐derived factor‐1, and matrix metalloproteinase 9 (MMP‐9).^41^ Additionally, the formation of the plastin‐3/plectin/cofilin complex enhances endothelial cell migration and lumen formation,^42^ while myosin‐9 is associated with cell proliferation, anti‐apoptosis, and angiogenesis.^43^

Serine proteases, particularly plasminogen activator–plasmin system, play an essential role in the remodeling of the extracellular matrix during angiogenesis.^44^ Plasminogen activator inhibitor helps maintain and remodel the extracellular matrix by inhibiting the conversion of plasminogen into plasmin.^44^ This is crucial for endothelial cell migration and vascular formation during wound repair. Pigment epithelium‐derived factor induces the regression of immature blood vessels formed during wound repair and aids in capillary refinement.^45^, ^46^ Consequently, pigment epithelium‐derived factor is associated with the resolution of the wound healing process and the restoration of tissue homeostasis after injury.^45^, ^46^ α2‐Macroglobulin is an acute‐phase protein that enhances vasculogenesis and angiogenesis by inducing nitric oxide generation and FGF‐2 expression.^47^

Endogenous ribonuclease inhibitor has been suggested to regulate angiogenin‐induced neovascularization,^48^ potentially promoting the growth of blood vessels.^48^ Thyroxine promotes angiogenesis by increasing bFGF mRNA expression through the integrin αvβ3/protein kinase D (PKD)/histone deacetylase 5 (HDAC5) signaling pathway.^49^ Thyroxine induces the phosphorylation of PKD and HDAC5, regulating the recycling of integrin αvβ3 for cell migration during angiogenesis.^49^ IQGAP1 mediates various biological processes, including growth factor signaling, cell extension, intercellular adhesion, cell migration, and phagocytosis.^50^ It interacts with flightless I, which regulates cell extensions for collagen tractional remodeling.^50^ Additionally, flightless I plays a role in the regulation of pericyte functions, including inflammation and angiogenesis.^51^

Extracellular matrix proteins in DFCM are crucial for the dynamic interaction among angiogenic cytokines, extracellular matrix proteins, and endothelial cells during angiogenesis.^52^ Fibrin and fibronectin, in particular, interact with extracellular matrix receptors in endothelial cells via αvβ3 integrin, potentially promoting angiogenesis and wound healing.^52^, ^53^ Semaphorin‐5A induces the proliferation and migration of endothelial cells while inhibiting apoptosis during angiogenesis.^54^ Additionally, Semaphorin‐5A increases MMP‐9, which aids in endothelial cell migration through extracellular matrix degradation.^54^ Hemoglobin subunit α, located at the myoendothelial junction of vessels, regulates endothelial nitric oxide synthase activity and nitric oxide diffusion.^55^ Apolipoprotein A1 exhibits anti‐angiogenic activity by inactivating extracellular signal‐regulated kinase 1/2 in hypoxic tissue environments.^56^ It also suppresses placental growth factor (PlGF), associated with pathological angiogenesis.^56^ Apolipoprotein D similarly demonstrates anti‐angiogenic activity and plays a role in axon regeneration and remyelination for functional recovery of peripheral nerve integrity.^57^

In DFCM, chloride intracellular channel protein 4 plays a crucial role in acidifying vacuoles during angiogenesis, facilitating endothelial cell tubulogenesis.^58^ EMILIN1 induces α4β1 integrin‐dependent adhesion and migration of various cell types.^59^ Moreover, EMILIN1 inhibits dermal and epidermal hyperproliferation, contributing to maintaining skin homeostasis.^59^ It also plays a role in elastogenesis during angiogenesis, maintains blood vascular cell morphology, and is a key regulator of lymphangiogenesis.^60^ In the category of protein modifying enzymes, hemopexin was identified and plays a critical role in removing excess free heme, which can be cytotoxic to endothelial cells. Moreover, it upregulates the expression of heme‐oxygenase‐1, a crucial factor in the recovery from ischemic tissue injury, thereby promoting angiogenesis.^61^ The 72 kDa type IV collagenase, also known as matrix metalloproteinase 2, is involved in cleaving the basement membrane and degrading interstitial matrix molecules, which enhances angiogenesis.^62^

Interstitial collagenase contributes to angiogenesis by its ability to degrade extracellular matrix components.^62^ Prothrombin is a key factor in the coagulation cascade, leading to thrombin formation, which in turn promotes angiogenesis and regulates blood coagulation.^63^ Matrix metalloproteinase 1 and thrombin promote angiogenesis by selective proteolytic activation of protease‐activated receptor‐1.^63^ Aminopeptidase N, also known as APN/CD13, has dual roles as both a cell surface receptor and a peptide‐cleaving enzyme. Its expression is associated with the migration of endothelial cells and fibroblasts during the wound healing process.^64^ Haptoglobin has been demonstrated to be involved in angiogenesis and vascular restructuring through the TGF‐β1‐ALK1‐Smad1/5 signaling pathway and PlGF.^65^ Coagulation factor X is a vitamin K‐dependent plasma protein that plays a crucial role in converting prothrombin into thrombin, promoting angiogenesis, and regulating blood coagulation.^63^, ^66^ Adipocyte enhancer‐binding protein 1 acts as a proinflammatory mediator and induces nuclear factor‐κB signaling. It has been suggested to promote angiogenesis by regulating aquaporin 1 and periostin genes.^68^ Complement C1r subcomponent and BMP1 are related to endothelial activities and functionally associated with the BMP2/TGF‐β/hedgehog signaling system.^69^

Lactadherin promotes VEGF‐dependent angiogenesis through interactions with αvβ3 and αvβ5 integrins, inducing Akt phosphorylation in endothelial cells.^70^ Peroxiredoxin family members are antioxidative cytoprotective enzymes. Peroxiredoxin‐1, which has also been identified as an endogenous ligand for toll‐like receptor 4, increases VEGF expression in endothelial cells.^71^ Peroxiredoxin‐1 induces HIF‐1α mRNA expression and enhances HIF‐1 activity, leading to increased VEGF expression.^71^ On the other hand, peroxiredoxin‐2 is involved in promoting angiogenesis by preventing oxidative inactivation of the VEGF receptor in endothelial cells.^72^ Peroxiredoxin‐4 induces the proliferation and migration of fibroblasts, accelerating wound healing and angiogenesis.^73^ Additionally, peroxiredoxin‐6 protects various cell types, including endothelial cells, from reactive oxygen species‐induced cytotoxicity and prevents wounded skin from ultraviolet damage, improving blood vessel integrity.^74^

Ectonucleotide pyrophosphatase/phosphodiesterase family member 2 is upregulated by bFGF and is involved in tubulogenesis for angiogenesis.^75^ Macrophage migration inhibitory factor presents pleiotropic functions, including proinflammatory cytokine and pro‐atherogenic factor. Furthermore, it has been demonstrated to promote angiogenesis by activating endothelial cells and recruiting endothelial progenitor cells.^76^ Peroxidasin, a multidomain heme peroxidase, plays a role in extracellular matrix stabilization.^77^ Peroxidasin 1 activates an ERK 1/2/Akt/focal adhesion kinase (FAK) pathway, inducing pro‐angiogenic genes and promoting angiogenesis.^77^ DDAH1 breaks down endogenous asymmetrical dimethylarginine, inhibiting nitric oxide synthesis.^78^ Additionally, DDAH1 is associated with endothelial cell motility and angiogenesis but not with vascular reactivity or hemodynamic regulation.^78^ HIF‐1α‐induced nitric oxide elevation enhances VEGF expression, promoting angiogenesis.^79^ Conversely, hypoxia‐induced increased arginase expression has been shown to inhibit nitric oxide production.^79^

Clusterin secretion is upregulated by VEGF, and secretory clusterin has been shown to be associated with endothelial cell proliferation, migration, and angiogenesis.^80^ Decorin interacts with various extracellular matrix proteins, including fibronectin, thrombospondin, tenascin, trophoelastin, and several collagen types, playing a critical role in extracellular matrix assembly.^81^ Decorin promotes cell adhesion, migration, and proliferation and regulates the activity of growth factors such as FGF‐2, TGF‐β, TNF‐α, PDGF, and insulin‐like growth factor. Additionally, decorin is linked to angiogenesis and extracellular matrix accumulation.^81^

Vinculin is a vital component of cell adhesion complexes associated with epithelial differentiation, adhesion strength, and stability.^82^ Mechanical tension in injured tissue stimulates the mechanosensitive protein α‐catenin, inducing vinculin expression to reinforce the endothelial barrier during angiogenesis.^83^ Fibulins, a family of secreted glycoproteins, modulate cell growth, motility, and adhesion.^84^ Fibulin‐1, in particular, suppresses tumor growth and progression by inhibiting angiogenesis and inducing apoptosis in endothelial and tumor cells.^84^

Plakoglobin inhibits the angiogenesis process by regulating endothelial cell migration and tube formation through its role in cell‐to‐cell adhesion.^85^ Protein kinase C activates the mitogen‐activated protein kinase (MAPK) pathway, promoting endothelial cell proliferation and playing a role in maintaining microcirculation through angiogenesis.^85^, ^86^ Extracellular matrix protein 1, a secreted glycoprotein, has been demonstrated to enhance cell proliferation, angiogenesis, and differentiation.^87^ Ras‐related protein Rab‐10 belongs to the Ras family of GTPases and regulates endoplasmic reticulum dynamics and morphology.^88^ Ras activation also leads to MAPK pathway activation, which is associated with vascular formation.^89^ However, the precise role of Rab10 in angiogenesis during wound healing requires further investigation.

## 5 CONCLUSION

In this study, our findings indicate that DFCM is enriched with numerous secretory proteins, which collectively form groups exhibiting significant protein–protein interactions that play pivotal roles in the regulation of angiogenesis. Importantly, the bioinformatic analyses conducted here shed light on the critical involvement of DFCM proteins in various stages of angiogenesis during the process of wound repair. Nonetheless, it is imperative that further investigations are undertaken to elucidate the precise roles of individual DFCM proteins or specific DFCM protein networks in the intricate regulation of angiogenesis.

## CONFLICT OF INTEREST STATEMENT

The authors declare no conflicts of interest.

## Data Availability

Data supporting the findings of this study are available upon request from the corresponding author. The data are not publicly available because of privacy and ethical restrictions.
